# Genomic Signature of the Natural Oncolytic Herpes Simplex Virus HF10 and Its Therapeutic Role in Preclinical and Clinical Trials

**DOI:** 10.3389/fonc.2017.00149

**Published:** 2017-07-14

**Authors:** Ibrahim Ragab Eissa, Yoshinori Naoe, Itzel Bustos-Villalobos, Toru Ichinose, Maki Tanaka, Wu Zhiwen, Nobuaki Mukoyama, Taishi Morimoto, Noriyuki Miyajima, Hasegawa Hitoki, Seiji Sumigama, Branko Aleksic, Yasuhiro Kodera, Hideki Kasuya

**Affiliations:** ^1^Cancer Immune Therapy Research Center, Graduate School of Medicine, Nagoya University, Nagoya, Japan; ^2^Department of Surgery II, Graduate School of Medicine, Nagoya University, Nagoya, Japan; ^3^Faculty of Science, Tanta University, Tanta, Egypt; ^4^Takara Bio Inc., Otsu, Japan; ^5^Department of Otolaryngology, Graduate School of Medicine, Nagoya University, Nagoya, Japan; ^6^Department of Transplantation and Endocrine Surgery, Graduate School of Medicine, Nagoya University, Nagoya, Japan; ^7^Office of International Affairs, Graduate School of Medicine, Nagoya University, Nagoya, Japan

**Keywords:** herpes simplex oncolytic viruses, genomic structure, HF10, talimogene laherparepvec, preclinical studies, combination therapy, clinical trials, future directions

## Abstract

Oncolytic viruses (OVs) are opening new possibilities in cancer therapy with their unique mechanism of selective replication within tumor cells and triggering of antitumor immune responses. HF10 is an oncolytic herpes simplex virus-1 with a unique genomic structure that has non-engineered deletions and insertions accompanied by frame-shift mutations, in contrast to the majority of engineered OVs. At the genetic level, HF10 naturally lacks the expression of *UL43, UL49.5, UL55, UL56*, and latency-associated transcripts, and overexpresses *UL53* and *UL54*. In preclinical studies, HF10 replicated efficiently within tumor cells with extensive cytolytic effects and induced increased numbers of activated CD4^+^ and CD8^+^ T cells and natural killer cells within the tumor, leading to a significant reduction in tumor growth and prolonged survival rates. Investigator-initiated clinical studies of HF10 have been completed in recurrent breast carcinoma, head and neck cancer, and unresectable pancreatic cancer in Japan. Phase I trials were subsequently completed in refractory superficial cancers and melanoma in the United States. HF10 has been demonstrated to have a high safety margin with low frequency of adverse effects in all treated patients. Interestingly, HF10 antigens were detected in pancreatic carcinoma over 300 days after treatment with infiltration of CD4^+^ and CD8^+^ T cells, which enhanced the immune response. To date, preliminary results from a Phase II trial have indicated that HF10 in combination with ipilimumab (anti-CTLA-4) is safe and well tolerated, with high antitumor efficacy. Improvement of the effect of ipilimumab was observed in patients with stage IIIb, IIIc, or IV unresectable or metastatic melanoma. This review provides a concise description of the genomic functional organization of HF10 compared with talimogene laherparepvec. Furthermore, this review focuses on HF10 in cancer treatment as monotherapy as well as in combination therapy through a concise description of all preclinical and clinical data. In addition, we will address approaches for future directions in HF10 studies as cancer therapy.

## Introduction

Oncolytic viruses (OVs) are currently being used effectively with therapeutic drugs to change the landscape of cancer treatment. OVs are considered immunotherapeutic targeted agents due to their selective replication within tumor cells and enhancement of the immune response. As a consequence, recent advances in viral genomics and tumor immunology have addressed OVs as a type of cancer therapy. To date, over 30 OVs belonging to seven DNA or RNA virus families have been successfully translated from preclinical studies to clinical trials (Table 1) (1). The Herpesviridae family includes human alphaherpesvirus-1 [Herpes simplex virus-1 (HSV-1)]. HSV-1 is the first human herpesvirus to be discovered and the most intensively investigated virus (2). The HSV family has common features, such as double stranded DNA (dsDNA) and an icosahedral capsid (3). The HSV family has taken precedence over other families in cancer treatment. For example, dlsptk, a type of HSV-1 virus, was the first OV to be engineered by deletion of HSV thymidine kinase (4). Talimogene laherparepvec (T-Vec, Imlygic™ formerly Oncovex^GM-CSF^), an HSV-1 virus encoding granulocyte macrophage colony-stimulating factor (GM-CSF), was the first OV approved by the US Food and Drug Administration for the treatment of melanoma (5).

**Table 1:** Families of oncolytic viruses (OVs).

	Herpes simplex virus-1 (HSV-1)	Adenoviruses	Paramyxoviruses		Poxviruses	Picornaviruses		Reoviruses	Rhabdoviruses	Retrovirus
**Family**	Herpesviridae	Adenoviridae	Paramyxoviridae		Poxviridae	Picornaviridae		Reoviridae	Rhabdoviridae	Retroviridae
**Genus**	Simplexvirus		Avulavirus	Morbillivirus	Orthopoxvirus	Enterovirus	Seneca virus	Orthoreovirus	Vesiculovirus	–
**Nucleic acid**	Double stranded DNA (dsDNA)	dsDNA	ssRNA	ssRNA	dsDNA	ssRNA	ssRNA	dsRNA	ssRNA	ssRNA
**OVs**	HF10 T-Vec G207 NV1020 HSV-1716 G47Δ M032	Telomelysin Ad5-CD/TKrep Ad5-D24-RGD Ad5-SSTR/TK Onyx-015 CGTG-102 INGN-007 (VRX-007) ColoAd1 CG7870/CV787 CG0070 Oncorine (H101) CG7060	**Newcastle Disease virus** PV701 MTH-68/H NDV-HUJ	**Measles virus (Edmonston)** MV-CEA MV-NIS	**Vaccinia (Wyeth strain)** JX-594	**Coxsackie virus (CVA21)** CAVATAK	Seneca Valley virus	**Reovirus (Dearing)** Reolysin	**Vesicular stomatitis virus (Indiana)** VSV-hIFNβ	Toca 511
					**Vaccinia (Western Reserve)** vvDD-CDSR	**Poliovirus (Sabin)** PVS-RIPO				
					**Vaccinia (Lister)** GL-ONC1 (GLV-h68)					

Most OVs, including the approved T-Vec, have been engineered to increase tumor selectivity and efficacy. HF10, on the other hand, is a spontaneously mutated virus without any insertion of foreign genes. The HF10 genome consists of linear dsDNA with a natural deletion of 6,127 kb and insertions of 6,027 bp accompanied by frame-shift mutations located at different nucleotide positions within the genome. These deletions and insertions caused a loss of expression of *UL43, UL49.5, UL55, UL56*, and latency-associated transcript (LAT) genes and overexpression of *UL53* and *UL54*. Many investigators have evaluated the effect of these deletions on the oncolytic characteristics of HF10 in different cell lines as well as tumor models of colon cancer, breast cancer, bladder cancer, pancreatic cancer, and melanoma. Preclinical studies have found that HF10: (a) has high innate tumor selectivity, (b) has high viral replication, (c) induces a complete cytopathic effect, (d) mediates a highly potent bystander effect, and (e) has potent antitumor efficacy against different malignancies. Consequently, preclinical studies have translated into successful clinical trials with promising results in different cancer types including recurrent metastatic breast cancer, recurrent head and neck squamous cell carcinoma (HNSCC), advanced pancreatic cancer, refractory and superficial cancers, and melanoma. Recently, there has been a lot of effort to establish the full layout of HF10 as an OV in cancer treatment. This review outlines a detailed approach for using HSV-HF10 as an OV. We will address the similarities and differences of the genomic structures of HF10, T-Vec, and other HSV OVs. Furthermore, we will describe the effect of the natural deletions in HF10 on its oncolytic efficacy in cancer treatment through a concise review of all preclinical studies and clinical trials, comparing it to genetically engineered viruses such as T-Vec. Finally, we will outline future directions for preclinical and clinical studies.

### HF10 Virion Structure

**Figure 1:**
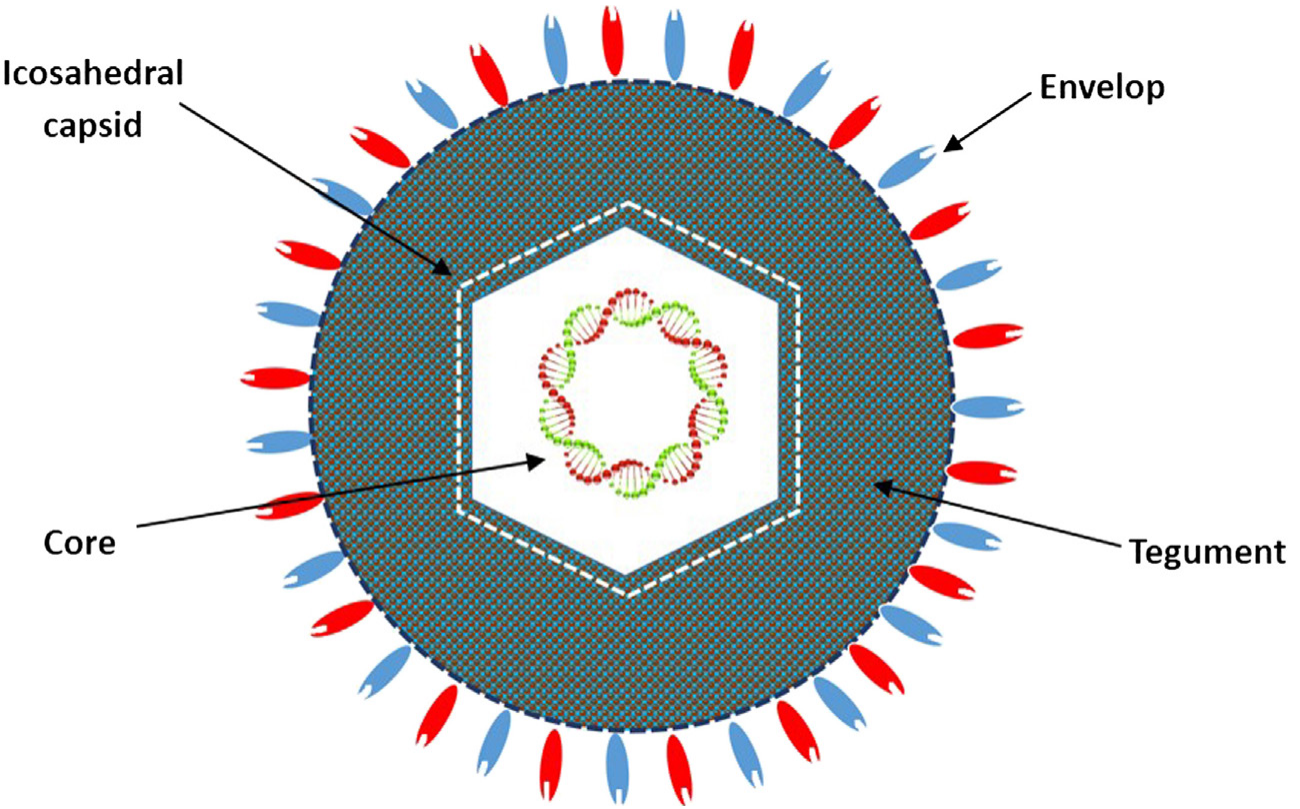
Diagrammatic structure of the HF10 virion. The HF10 virion is composed of four elements: *envelope*, contains glycoprotein receptors; *Tegument*, consists of viral proteins; *Icosahedral capsid*, comprised of capsomers and a nucleocapsid in the outer layer; and *Core*, contains linear double stranded DNA.

HF10 was originally purified from the HSV-1 strain HF as HF clone 10 (HF10) (6). The HF10 virion is similar to other HSV-1 virions. Early studies revealed that the HSV virion consists of four elements as shown in Figure 1: (a) a core containing linear dsDNA wrapped as a toroid or spool with the negative charges of DNA neutralized by polyamines (spermine and spermidine); (b) an icosahedral capsid comprised of 162 capsomers arranged in a *T* = 16 symmetry containing a nucleocapsid in the outer layer composed of four viral proteins (VP) plus VP5 as the major capsid protein; (c) a tegument consisting of an unstructured proteinaceous layer surrounding the capsid composed of 18 VP with VP16 as the most notable; and (d) an envelope, consisting of glycoproteins gB, gC, gD, gE, gG, gH, gI, gK, gL, and gM (7, 8). HF10 lacks *UL49.5* that encodes gN, which links with gM (UL10 protein) to form a disulfide-linked complex (9). Moreover, HF10 overexpresses *UL53*, which encodes gK, a regulator of the egression process of the HSV virion from infected cells (10).

## Genomic Structures of HF10 and T-Vec

**Figure 2:**
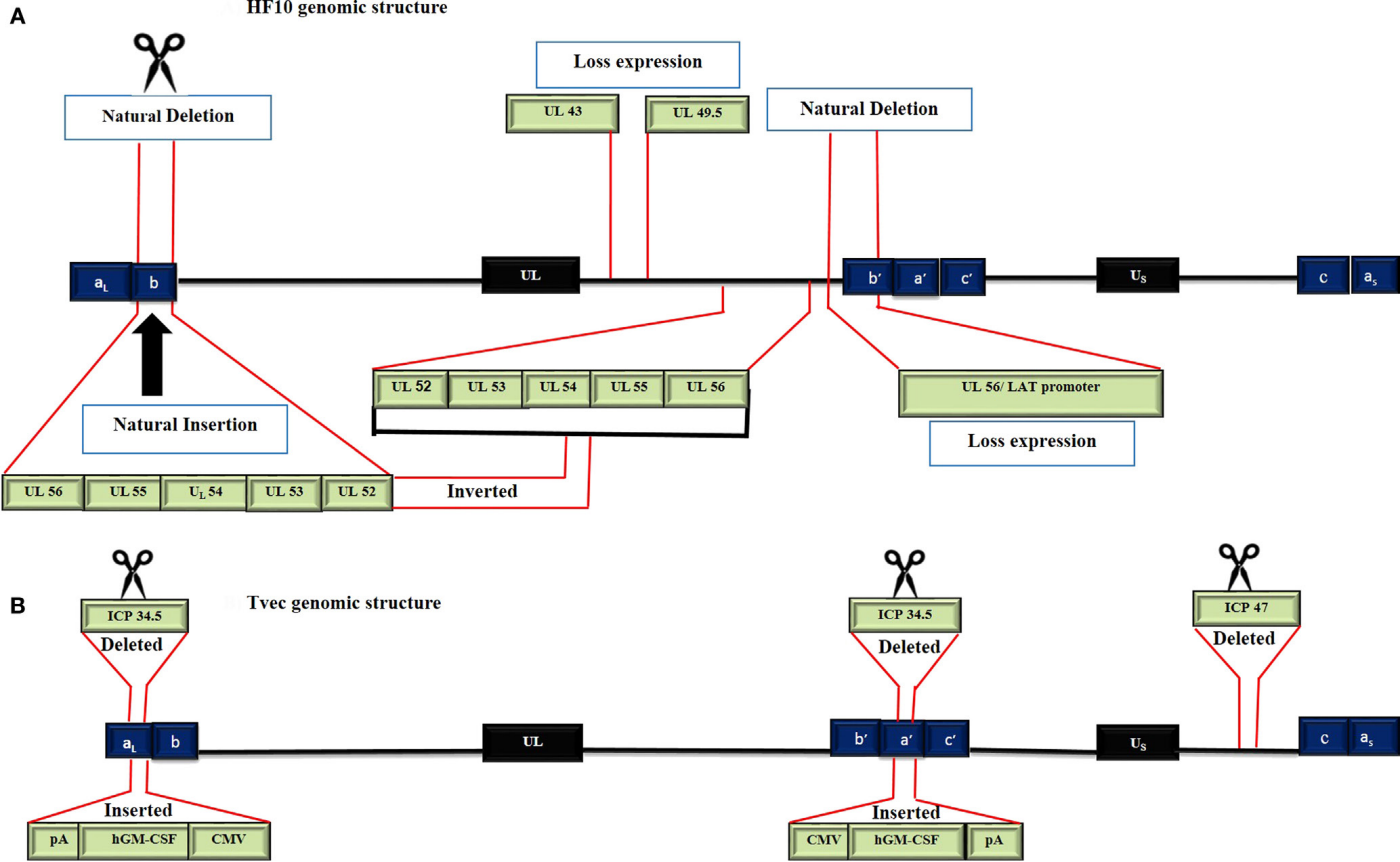
Genomic structure of HF10 and talimogene laherparepvec. Each genome consists of two components: a Unique Long sequence (UL) flanked by a terminally repeated long sequence (TRL) and an internally repeated long sequence (IRL) and a Unique short sequence (US) flanked by a terminally repeated short sequence (TRS) and an internally repeated short sequence (IRS). **(A)** The HF10 genome has two deletions: 3,832 bp were deleted at the *UL56*/IRL junction and 2,295 bp were deleted in the TRL region and replaced by 6,027 bp of *UL56, UL55, UL54, UL53*, and *UL52*. A frame-shift mutation led to the loss of *UL43, UL49.5*, and *UL55* expression. **(B)** T-Vec genomic structure. *ICP47* was deleted. Both copies of *ICP34.5* were deleted and replaced with an h*GM-CSF* cassette that is composed of immediate early promoters from cytomegalovirus (CMV) and polyadenylation signal (pA) (bovine growth hormone).

To date, there are 17 strains of HSV-1 that have been isolated (ICTV 2015 taxonomy). Seven genomes have been completely or partially sequenced. HSV HF17 (NC_001806, X14112) is often used as a reference for genome sequence comparison (11). The HF10 genome was the first HSV genome to be completely sequenced, while T-Vec has only been partially sequenced. The HF10 and T-Vec genomes have the following similarities (Figure 2). (a) Both genomes are made up of linear dsDNA. (b) Each genome is composed of two unique inverted sequences, a unique long sequence (UL) flanked by a terminally repeated long sequence [TRL = (a_L_, b)], and an internally repeated long sequence [IRL = (b^/^a^/^)]. (c) Each genome also has a unique short sequence designated as (US) bracketed by a terminally repeated short sequence [TRS = (c, a)], and an internally repeated short sequence [IRS = (c^/^a^/^)] (12, 13).

HF10 differs from T-Vec in their strain origins and their genomic deletions and insertions panel (Table 2). T-Vec was genetically modified from the JS1 strain to improve tumor-selective replication and immune response. As shown in Figure 2B, both copies of the *ICP34.5* gene have been deleted from the parent virus genome to suppress its replication in normal tissues. The *ICP47* gene has also been deleted to increase the expression of MHC class I on infected cells. Moreover, the hGM-CSF cassette has been inserted in lieu of the *ICP34.5* gene loci to enhance the antitumor cytotoxic immune response. Expression of hGM-CSF is derived from the cytomegalovirus and polyadenylation signal (pA) (bovine growth hormone) immediate early promoters, respectively (13).

**Table 2:** Genomic comparison of HF10 and other herpes simplex virus-1 oncolytic viruses (OVs).

OVs	HF10	Talimogene laherparepvec	G207	NV1020	G47Δ	HSV-1716	Mo32
**Strain**	HF strain	Js1 strain	F strain	F strain	F strain	17+ strain	F strain
**Isolation or generation year**	1991	2003	1995	2002	2001	1991	2000
**DNA sequencing**	Completely	Partially	Partially	Partially	Partially	Partially	Partially
**Genetic manipulation**	Natural deletions and insertion	Genetically modified	Genetically modified	Genetically modified	Genetically modified	Genetically modified	Genetically modified
**Deleted Genes**	UL56 Latency-associated transcript (LATs)	ICP 34.5 ICP 47	ICP34.5 ICP6	ICP34.5 ICP4 ICP0	ICP34.5 ICP6 ICP 47	ICP34.5	ICP34.5
**Inserted genes**	UL52, UL53, UL54, UL55	hGM-CSF or mGM-CSF	LacZ		–	–	IL12
**Overexpression**	UL53, UL54	–	–	Thymidine kinase	–	–	–
Loss of expression	UL43, UL49.5, UL55, UL56, LAT	–	–	UL24	–	–	–

HF10 has natural deletions and insertions within the genome. The *UL56*/IRL junction has been deleted from 116.515 bp to 120.346 bp, leading to the lack of expression of *UL56* and LATs (Figure 2A). In addition, 2,295 bp of the TRL has also been deleted and replaced by 6,027 bp that express the *UL52_partial_, UL53, UL54, UL55*, and *UL56* inverted sequences. Both deletions and insertions lead to duplicated copies of *UL53, UL54, UL55*, two incomplete copies of *UL56*, and one complete and one incomplete copy of *UL52*. Frame-shift mutations in the N-terminal region cause a loss of the functional expression of the *UL43* and *UL49.5* gene products (12).

As noted above, the deletions cause the gene products of *UL43, UL49.5, UL55, UL56*, and LAT to not be expressed, whereas duplication leads to *UL53* and *UL54* overexpression. The main question here is “What are the functions of the deleted and duplicated genes and their effect on HF10 antitumor efficacy?” To answer this question, the function of the deleted genes must be known.

### *UL43*: (γ Gene, Accessory Gene)

The HSV-1 *UL43* gene acts as a γ accessory gene. *UL43* mRNA encodes a hydrophobic transmembrane protein (14) that is conserved in the α and γ herpesviruses but absent in β herpesviruses (15–17). The UL43 protein is dispensable for viral growth in cell culture. Deletion of UL43 does not impair characteristics including virus entry, cell–cell fusion *in vitro*, viral replication *in vivo*, or neuroinvasiveness (18). Another study mentioned that HSV17*^UL43^*^−^ has the ability to infect 40 to 60% of dendritic cells *in vitro* but the role of this deletion remains unclear (19). Thus, the lack of *UL43* expression may play a role in the direct interaction between HF10 and antigen-presenting cells (dendritic cells) to enhance the immune response.

### *UL49.5*: (γ, Core Gene)

The *UL49.5* gene is a γ core gene that is conserved in all HSVs. It encodes a type 1 transmembrane glycoprotein N (gN). This gN forms a heterodimeric complex with glycoprotein M (gM) (20, 21). *UL49.5* homologs of HSV-1 have no effect on the transporter associated with antigen processing function (TAP) (20, 22). Hence, *UL49.5* deletion is likely involved in the syncytial (syn) phenotype of HF10 while the effect of this deletion on the oncolytic capacity of HF10 remains unclear.

### *UL53*: (γ, Accessory Gene)

*UL53* encodes glycoprotein K (gK) protein. gK regulates HSV egression from infected cells. gK is the most common locus of syn mutations. HF10 has duplicated *UL53*, which leads to gK overexpression, which causes accumulation of virus in the perinuclear space of infected cells as long as there are defects in viral egression (10). The accumulation of virus in cells accounts for a margin of safety when HF10 is inoculated into humans, as no shedding of virus to other organs has been observed. Previous studies have reported that gK prevents the formation of syncytia (23, 24). However, HF10 forms complete syncytia *in vitro* in different cell lines.

### *UL55*: (γ, Accessory Gene)

*UL55* acts as a γ accessory gene. *UL55* mRNA encodes a non-structural protein that is associated with sites of virion assembly. Previous studies have shown that *UL55* is not necessary for intraperitoneal virulence and establishment of latency in mice (25, 26).

### *UL56*: (γ, Accessory Gene)

The *UL56* gene is located at the right end of the unique long region of the HSV-1 genome (26). During acute infection, HSV-1 *UL56* is naturally expressed; it is considered a component of the HSV-1 virion (27). *UL56* is involved in the pathogenicity and latency of HSV-1. Lack of *UL56* expression may be involved in viral neuroinvasiveness (28). A previous study has reported that the deletion of *UL56* from the HSV-1 strain HFEM is pathogenic in tree shrews (29).

### Latency-Associated Transcripts

Latency-associated transcripts are expressed during virus latency. LATs play a role in neuroinvasiveness and reactivation from latency. One study has reported a correlation between LATs and *ICP34.5* deletion compared with wild-type virus. LATs alone and *ICP34.5* alone each reduced spontaneous reactivation by 10–30% and 10%, respectively, compared to wild type. However, deletion of both LATs and *ICP34.5* led to undetectable levels of reactivation, even when the amount of virus was increased to 10^8^ pfu (30). Therefore, the lack of LATs in the HF10 genome leads to suppression of reactivation from latency and supports the safety margin in the long-term, after treatment.

### Genomic Deletions and Insertions in HF10 and Other HSV OVs

The identification of viral genes provides a strategy for genetically modifying OVs. To date, there are seven HSV-1 OVs (Table 2) being investigated in clinical trials. When we compare the genomic structure of OVs, we can see that the deletions and insertions of genes in HF10 are different from those in other OVs. *ICP34.5* is deleted in HSV-1 OVs used in clinical trials, but present in HF10. *ICP34.5* is thought to be involved in HSV neurovirulence. However, the exact mechanism by which HSVs induce encephalitis is unclear (31). HSV-1 OVs are classified according to the number of modified genes. First-generation OVs have only one modified gene (*ICP34.5* deletion), such as HSV-1716 [*ICP34.5*(−)] (32). Second-generation OVs have several gene deletions or insertions, and include OVs, such as HF10, NV1020 [*ICP34.5*(−), *ICP4*(−), *ICP0*(−), *TK*(+)] (33), G207 [*ICP34.5*(−), *ICP6*(−), *LacZ*(+)] (34), and G47Δ [*ICP6*(−), *ICP34.5*(−), *ICP47*(−)] (35). Third-generation OVs include therapeutic genes such as T-Vec [*GM-CSF*(+)/*ICP34.5*(−)/*ICP47*(−)] (13), and Mo32 [*ICP34.5*(−)/*IL12*(+)] (36).

## Preclinical Studies of HF10 as Monotherapy

**Figure 3:**
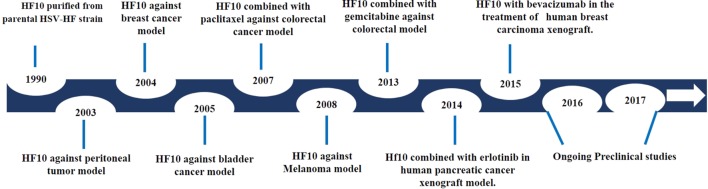
History of HF10 preclinical studies. Antitumor efficacy of HF10 against tumor models in previous years.

After investigating genomic changes in HF10, many investigators evaluated the oncolytic effect of HF10 in different malignant tumor models (Figure 3). Preclinical studies were conducted to evaluate the effect of HF10 replication on tumor selectivity and antitumor efficacy. HF10 was evaluated *in vitro* against Colon 26 and melanoma B16 cell lines, which showed that HF10 VP mediate cell–cell fusion to form enlarged multinucleated cells (syncytia formation). Furthermore, the therapeutic efficacy of HF10 was studied in murine and human breast cancer *in vitro* animal models. HF10 was also investigated in human and murine bladder cancer cell lines and in disseminated peritoneal metastasis.

To compare the antitumor effect and genomic structure of OVs, HSV-1 hrR3 *[ICP6(*−*)]* was chosen as a control due to the deletion of the *UL39* gene. *UL39* is also deleted in G207 and G47Δ HSV OVs. *UL39* encodes ICP6, the large subunit of ribonucleotide reductase, which is required for viral replication in non-dividing cells (37, 38). However, the deletion of *UL39* was postulated to increase the tumor selectivity of hrR3. HF10 induced complete syncytia formation in Colon 26 and melanoma B16 cell lines *in vitro*, while hrR3 induced a partial cytopathic effect. Furthermore, in a peritoneal tumor model, injection of 1 × 10^7^ pfu of HF10 showed a more potent antitumor response, with a long-term survival rate over 90 days, than the same dose of hrR3 (39). Two studies have confirmed that HF10 replication was higher than hrR3 replication by 10-fold in CT26 cell and NfSa Y83 fibrosarcoma cells (40, 41). One limitation of hrR3 was elevated levels of neutralizing antibodies against hrR3 after 5 days of intraperitoneal inoculation (42). In addition, Luo et al. reported that HF10 has a greater bystander effect than hrR3 due to enhanced expression of connexin 43 subunits (43). Regarding other genetic deletions, Oncovex*^ICP34.5^*^(−),^*^ICP47^*^(−)^ induced a classic cytopathic effect against human cell lines including HT1080 (fibrosarcoma), HCT 116 (human colonic carcinoma), CAPAN-1 (human pancreatic adenocarcinoma), and BHK (hamster normal baby kidney) cells (44). Therefore, natural deletions in HF10 may increase tumor selectivity, replication, cytopathic effect, and bystander effect compared with known deletions in other HSV OVs.

HF10 viral replication and cytotoxicity has also been studied in human and mouse melanoma cell lines (G-361 cells and clone M3 cells). HF10 induced 100% cell lysis in the clone M3 cell line after 48 h at MOI 3 and 0.3. Even at MOI 0.03, 92.6% of melanoma cells were lysed 72 h after infection (45). T-Vec was studied in the SK-MEL-28 melanoma cell line. At MOI 0.1 and 1, T-Vec caused 48 and 89% cell death, respectively, after 24 h. At 48 h after infection, T-Vec induced 84 and 100% cell death, respectively (EMEA*/*H*/*C*/*002771/0000). In an *in vivo* study, HF10 significantly reduced tumor growth in a subcutaneous melanoma model. Complete survival was shown in an intraperitoneal melanoma model without any obvious adverse effects. The antitumor efficacy and safety of HF10 were supported by detection of HF10 antigens with lymphoid cells and polymorphonuclear cells for at least 7 days after treatment (45). Recently, the B16F10 melanoma cell line, which lacks the expression of HSV entry receptors, was modulated to express the HSV-1 entry receptor Nectin1. A preliminary result with T-Vec showed sensitivity against Nectin1-expressing B16F10 *in vitro* and prolonged survival in an *in vivo* model (46).

Studies were extended to determine HF10 cellular tropism in other tumor models. The therapeutic efficacy of HF10 was studied in murine and human breast cancer animal models. HF10 efficiently replicated with high cytolytic effect in human and mouse breast cancer cell lines (MCF-7 and YMB-1, respectively). After 48 h, HF10 lysed almost all cells at MOI 3 and 0.3. However, HF10 replicated poorly in the MM102-TC mouse breast cancer cell line, even with increasing MOI titers. With inoculation of 1 × 10^7^ pfu of HF10, there was suppression of tumor growth with prolonged survival rates up to 120 days without any neurologic or toxic side effects (47). In another study, HF10 and T-Vec were evaluated in a human breast adenocarcinoma cell line (MDA-MB-231). At MOI 1, HF10 caused approximately 50 and 90% cell death after 24 and 48 h post infection, respectively (48), while at the same MOI, T-Vec induced only 13.6 and 64.4% cell death after 24 and 48 h post infection, respectively (EMEA*/*H*/*C*/*002771/0000). In summary, HF10 with natural deletions had a significant oncolytic effect against human breast cancer cell lines.

Furthermore, the oncolytic effect of HF10 was investigated in human and murine bladder cancer cell lines (T24 and MBT-2) *in vitro* and also in a disseminated peritoneal metastasis model and a bladder cancer model. At MOI 3, HF10 replicated well in both T24 and MBT-2 cell lines and induced complete cell death by 48 h. In addition, serial HF10 treatments significantly prolonged survival rates in both models. HF10 safety and selectivity were supported by the presence of HSV antigens in the bladder on day 1 after intravesical treatment without shedding to other organs (49). These results suggested that HF10 has promising effects in a bladder cancer model and should be studied in a clinical trial. Chemotherapeutic or immunotherapeutic agents have not yet been approved for the treatment of bladder cancer due to the lack of effectiveness. Among OVs, only one adenovirus CG0070*^GM-CSF^*^(+)^ phase II/III study is ongoing (NCT01438112); a durable response was observed in a phase I study (50).

Taken together, all the preclinical data on HF10, such as the loss of *UL56*, LATs, *UL43*, and *UL49.5* expression and *UL53* overexpression from the HF10 genome, lead to the following characteristics: innately high tumor selectivity, high viral replication, complete cytopathic effect, mediation of a highly potent bystander effect, and potent antitumor efficacy.

## Preclinical Studies of HF10 as Combination Therapy

To date, OVs have not shown serious toxicities or any therapeutic resistance, in contrast to chemotherapeutic drugs that cause severe dose-limiting toxicities and emerging cell resistance. As each approach has different mechanisms of action, combination therapy with OVs and chemotherapy enhances the antitumor effect with limited toxic side effects. A number of chemotherapeutic drugs are able to modulate the activities of myeloid-derived suppressor cells (MDSCs) and regulatory T cells in the suppressive tumor microenvironment. Gemcitabine (GEM) inhibits MDSCs and enhances antitumor immune responses through T cell expansion (51). To date, GEM has been extensively investigated in combination with many OVs in different malignancies, including pancreatic cancer (52–56), renal cell carcinoma (57), and lung cancer (58, 59). Esaki et al. evaluated the synergistic effect between HF10 and GEM in a bilateral colorectal cancer model. After 3 days of GEM treatment, HF10 was injected at a dose of 1 × 10^7^ pfu for 3 days to avoid possible interference with its replication. The study showed complete reduction of tumor size when HF10 was injected on the same side or even on the contralateral side. The oncolytic effect was enhanced by a significant decrease in CD11b^+^/F4/80^+^ macrophages and CD11b^+^/Gr-1^+^ MDSCs after GEM injection (60). GEM is one of the first-line therapeutic agents against pancreatic carcinoma with a median survival rate 4.4–5.6 months (61, 62). Unfortunately, combination therapy with other cytotoxic agents produced intolerable toxicities without any added benefits. In contrast, HF10 had a promising antitumor effect with a high safety margin in the investigator-initiated clinical studies for pancreatic cancer. Hence, HF10 will be an ideal agent to combine with GEM to achieve a high antitumor effect against pancreatic cancer with minimal side effects.

Regarding other chemotherapeutic drugs, paclitaxel induces cell death through mitotic arrest due to its effect on microtubule stabilization (63). HF10 has been combined with paclitaxel to enhance antitumor efficacy in *in vitro* and *in vivo* immunocompetent colorectal cancer models. Paclitaxel did not interfere with the replication or cytotoxicity of HF10 with CT26 cells *in vitro*. Paclitaxel and HF10 combination therapy resulted in superior survival rates in peritoneal colorectal cancer compared with either treatment alone (40). High proportions of mitotic and apoptotic cells were reported in combination with Reovirus type 3 Dearing strain (ReoT3D) OV and paclitaxel in non-small cell lung cancer cells (58). Another study investigated combination therapy with paclitaxel plus oncolytic Rhabdovirus Maraba MG1 virus in breast cancer, which showed controlled tumor growth and prolonged survival (64).

As with other OVs, the antitumor activity of HF10 depends on two mechanisms of action: selective replication within tumor cells causing tumor cell bursting and spreading and expression of tumor antigens, which induce an antitumor immune response (65). Erlotinib, an epidermal growth factor receptor (EGFR) tyrosine kinase inhibitor, binds to the ErbB-1 receptor, thus inhibiting tyrosine kinase activity and disrupting the activity of downstream pathways, including the Ras/Raf mitogen-activated protein kinase, phosphoinositide-3 kinase/Akt, and Jak2/STAT3 pathways (66). In addition to inhibiting cell proliferation, erlotinib also induced apoptosis and anti-angiogenesis of tumor cells (67). Previous studies have reported that human pancreatic cancer cell lines BxPC-3 and PANC-1 cells express EGFR (68, 69). Yamamura et al. evaluated the antitumor efficacy of HF10 combined with erlotinib in human pancreatic xenograft *in vitro* and *in vivo* using BXPC-3 and PANC-1 (70). The study reported that HF10 induced cell lysis in both cell lines; however, erlotinib was only sensitive in BxPC-3 cells. Combination treatment with HF10 and erlotinib resulted in a more significant cell lysis effect in BxPC-3 cells than with either HF10 or erlotinib alone. In BxPC-3 subcutaneous xenograft models, HF10 alone suppressed tumor growth more than erlotinib alone. However, in combination therapy, erlotinib caused high distribution of HF10, resulting in a significant tumor growth reduction compared with HF10 alone. Interestingly, the survival rate with HF10 alone was longer than with erlotinib alone (70).

The most important obstacle for OVs is the elevation of interstitial fluid pressure within tumors, which directly affects viral distribution (71). HSVs induce vascular endothelial growth factor (VEGF) production, which enhances angiogenesis in cells (72, 73). Bevacizumab is a monoclonal antibody suppressing tumor angiogenesis through inhibition of VEGF-A, which has been shown to be overexpressed in different solid tumors (74, 75). Tan et al. examined the oncolytic activity of HF10 in combination with bevacizumab in an experimental human breast carcinoma xenograft model (48). They showed that the MDA-MB-231 human breast cancer cell line has higher VEGF-A expression than the MC7 and T47D cell lines. By increasing MOI and time, HF10 alone induced cell cytotoxicity in the MDA-MB-231, MC7, and T47D cell lines. Bevacizumab did not induce any cell toxicity or interference with HF10 replication. In this study, two tumor models were established in BALB/c Slc-nu/nu mice bearing a single subcutaneous tumor or an advanced subcutaneous tumor. Intratumoral inoculation of HF10 (10^6^ pfu) and bevacizumab (5 µg i.p.) significantly inhibited tumor growth in both models. In addition, immunohistochemical studies showed that the combination of HF10 and bevacizumab replicated more efficiently and with syncytia formation than HF10 treatment alone. More upregulation of VEGF-A with downregulation of CD31 was observed in endothelial cells after treatment with bevacizumab and HF10 compared with HF10 alone in both the single and advanced subcutaneous tumor models (48). A similar effect of bevacizumab was reported with other OVs, including adenoviruses (76), hrR3 (77), vaccinia virus (78), and reovirus (79).

## HF10 Clinical Trials

### Phase I Clinical Trial in Breast Cancer

**Figure 4:**
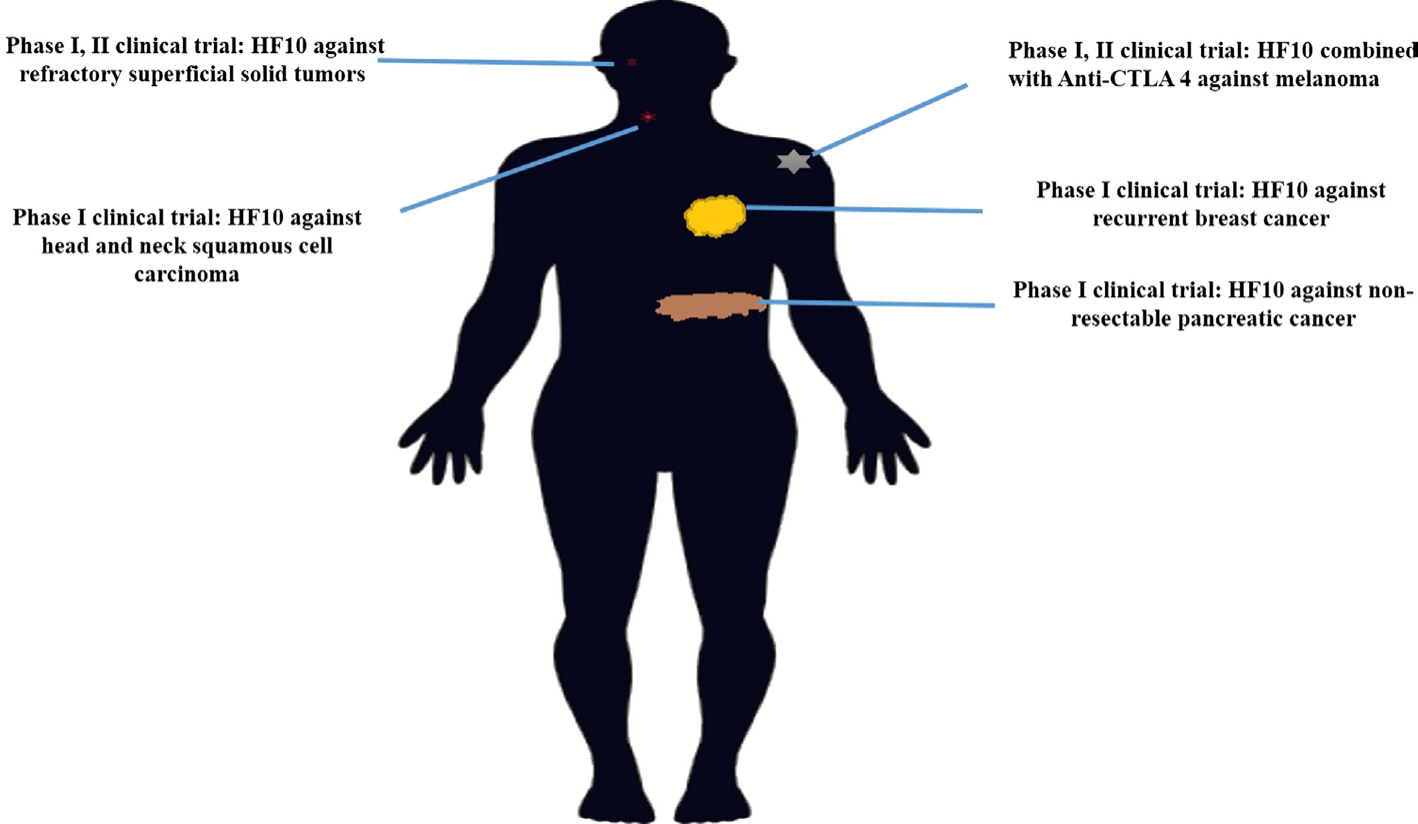
History of HF10 clinical studies. Clinical trials of HF10 of various phases against different malignancies in previous years.

HF10 has transitioned from preclinical to clinical trials to evaluate its therapeutic effect on human malignancies (Figure 4). The first clinical trial was performed from 2003 to 2006 by a team that included the surgery II, virology, and histopathology departments at the Graduate School of Medicine, Nagoya University, in Japan. The phase I clinical study evaluated the toxicity and efficacy of HF10 when directly injected intratumorally into cutaneous or subcutaneous metastatic nodules of recurrent breast cancers. All six patients had undergone mastectomy with recurrence after conventional therapies including chemotherapy, hormonal therapy, radiotherapy, and surgery. Patient age ranged from 48 to 76 years. They were seropositive for HSV and had metastatic recurrence in the skin (6/6), lymph nodes (4/6), lung (2/6), brain (1/6), and bone (1/6). In addition, all patients had more than 10 cutaneous and subcutaneous nodules. The first nodule was injected with diluted HF10, with doses ranging from 1 × 10^4^ to 5 × 10^5^ pfu/0.5 mL for 3 days. Another nodule was injected with sterilized saline as a control (Table 3). All patients tolerated the treatment well without any serious adverse effects. Histological examination showed nuclear viral inclusion bodies and adequate HF10 replication with high selectivity and distribution within malignant cells only. Tumor cell deformation was observed histologically, with 30 to 100% tumor death. Interestingly, a wide range of melting like fibrosis was observed after tumor cell destruction. There was considerable cytotoxic CD8^+^ T cell infiltration around tumor islets. Moreover, there was no change in the count of blood cells such as white blood cells and natural killer (NK) cells, or in the levels of cytokines, such as IL10, IL12, IFNα, and IFNβ. These data supported HF10 safety through selective replication within tumor cells without any severe side effects. Furthermore, HF10 induced a cytotoxic immune response against breast cancer with CD4^+^ and CD8^+^ T cell infiltration (80, 81).

**Table 3:** Profiles and responses of patients with metastatic breast cancer in a HF10 phase I clinical trial.

Patient No.	Age (years)	Recurrence region	Prior therapy	HF10 pfu/0.5 mL × 3 days	No. of Doses	Response	Side effects	Shedding
1	61	Skin, LN, lung, brain	CT-, HT-, RT-	1 × 10^4^	1	Moderate response	None	No shedding into body fluids
2	62	Skin, LN	CT-, RT-	2 × 10^5^	1	Mild response		
3	48	Skin, LN, lung, bone	SR	3 × 10^5^	3	Marked response		
4	66	Skin, LN	CT-, HT-	5 × 10^5^	1	Moderate response		
5	72	Skin	S-, CT-, HT-	5 × 10^5^	3	Complete response		
6	76	Skin	CT-, HT-	5 × 10^5^	3	Not applicable		

*CT, chemotherapy; HT, hormonal therapy; RT, radiotherapy; SR, surgery*.

On the other hand, in the first clinical trial of T-Vec in 30 patients with cutaneous or subcutaneous metastases, the OV was injected into cutaneous or subcutaneous nodules in 14 breast cancer patients. Age ranged from 39 to 80 years; half of the patients were HSV seropositive and the other half were seronegative. T-Vec doses ranged from 10^6^ to 10^8^ pfu/mL in 1 or 3 injections. In this study, there was no complete or even partial responses, but stable disease was observed without significant differences between seropositive or seronegative patients. Most patients tolerated the treatment well, with some side effects such as grade I pyrexia, low-grade anorexia, nausea, fatigue, and vomiting (82).

### Phase I Clinical Trial in HNSCC

An additional study demonstrated the safety and efficacy of HF10 in a phase I dose-escalation pilot study at the School of Medicine, Nagoya University, in Japan, on February 12, 2005. Two patients with advanced HNSCC were HSV seropositive. They were classified with aT2N1M0 disease, with several skin metastases and rT0N3M1 disease, with lymph node and skin metastasis, respectively (Table 4). Adverse effects, virus replication, and immunological response were evaluated after intratumoral injection of HF10 (1 × 10^5^ pfu/1 mL or 0.5 mL for 3 days). In both patients, HF10 replicated well and induced tumor cell death with significant CD4^+^ or CD8^+^ cell infiltration. The patients had a low-grade fever after injection but no other obvious adverse effects. As no significant regression in tumor size was observed on days 13 and 15 after treatment, higher doses of HF10 might be used in another trial (83). Five patients with positive or negative HSV serotype that had metastatic head and neck cancer received three doses of Oncovex*^GM-CSF^* (10^6^, 10^7^, and 10^8^ pfu/1 mL) for 3 days. Stable disease was observed without any complete or partial response. Some side effects such as pyrexia, low-grade anorexia, nausea, fatigue, and vomiting were observed (82).

**Table 4:** Profiles and responses of patients with metastatic HNSCC in a HF10 phase I clinical trial.

Patient	Age (years)/sex	Clinical stage	Prior therapy	HF10 pfu/mL × 3 days	Time	Response	Side effects
1	79/female	rT0N3M1	CT, RT	10^5^ pfu/0.5 mL	1	No significant tumor regression on day 13 or 15	Low-grade fever after injection only
2	64/male	rT0N3M1	CT, SR	10^5^ pfu/1 mL	1		

*HNSCC, head and neck squamous cell carcinoma; CT, chemotherapy; RT, radiotherapy; SR, surgery*.

### Phase I Clinical Trial in Pancreatic Cancer

A phase I clinical trial was performed in eight male patients with invasive pancreatic ductal carcinoma from 2005 to 2009 at the School of Medicine, Nagoya University, in Japan. All eight patients were HSV seropositive because of safety concerns. Six patients received one injection of HF10 (1 × 10^5^/two patients, 5 × 10^5^/one patient, and 1 × 10^6^/three patients) per day for three consecutive days. After 3 days of injections, the patients were given no further treatment for 30 days and monitored for adverse and therapeutic effects. The first dose of 0.5 mL was injected in four sites or as 2.0 mL during laparotomy. The other two doses were injected using an intratumoral catheter inserted at the time of surgery. Moreover, the last two patients received an additional injection of 10^6^ pfu/1.0 mL HF10 once a week for total of 3 weeks *via* endoscopic ultrasound (Table 5). All patients tolerated the treatment well without any observed adverse effects after treatment. Three patients showed declines in the tumor marker CA19-9. There was no HSV shedding into the blood or body fluids based on plaque-forming assays at this time. HF10 envelope protein was also detected in autopsy specimens with infiltrations of macrophages, CD4^+^ and CD8^+^ cells, and activation of NK cells, suggesting that HF10 enhances antitumor immunity. The response to treatment was classified as stable disease in three patients, partial response in one patient, and progressive disease in four patients. Survival time ranged from 98 to 318 days, with an average of 180 days. These results suggested that higher doses of HF10 can be used in future trials (84, 85).

**Table 5:** Profiles and responses of patients with metastatic pancreatic cancer in a HF10 phase I clinical trial.

Patient	Age (years)	Clinical stage	HF10 PFU/0.5 mL/days	Time	Response	Survival (days)	Side effects	Shedding
1	68	Invasive ductal carcinoma	1 × 10^5^ × 3	1	PD	200	None	No shedding into body fluids
2	61		1 × 10^5^ × 3	1	SD	166		
3	60		5 × 10^5^ × 3	3	SD	318		
4	52		1 × 10^6^ × 3	1	PD	98		
5	73		1 × 10^6^ × 3	3	PR	209		
6	76		1 × 10^6^ × 3	3	SD	315		
7	49		1 × 10^6^ × 6	6	PD	206		
8	64		1 × 10^6^ × 6	6	PD	113		

*PD, progressive disease; SD, stable disease; PR, partial response*.

### Phase I and Phase II Clinical Trials in Refractory Superficial Cancers and Melanoma in the US

A phase I clinical trial in patients with refractory superficial cancers and melanoma was conducted at the University of Pittsburgh in the United States. This trial evaluated the tolerability and efficacy of HF10 therapy in 26 patients, including HSV seropositive and seronegative patients, with refractory superficial cancers and melanoma. The trial was divided into two stages. In Stage 1, patients received a single HF10 dose at 1 × 10^5^, 3 × 10^5^, 1 × 10^6^, or 1 × 10^7^ pfu. In Stage 2, patients received four injections of HF10 at 1 × 10^6^ to 1 × 10^7^ pfu. The results showed that adverse events of any kind occurred in 34.6% of patients overall. Drug-related adverse events included chills (11.5%), fatigue (7.7%), pyrexia (3.8), and injection site reaction (6%). In comparison, T-Vec caused pyrexia (52%), fatigue (48%), and nausea (30%) in 50 melanoma patients. Moreover, no significant difference was observed between HSV-1 seropositive and seronegative patients. In summary, HF10 was safe and well tolerated. The response rate was evaluated in 24 patients. Eight patients had stable disease. The reduction in tumor size in some patients ranged from 30 to 61%. Interestingly, one patient showed pathological complete response after 4 months of treatment (86).

A phase II clinical trial of HF10 combination therapy was conducted in the United States. HF10 was combined with ipilimumab (anti–CTLA-4) in patients with unresectable or metastatic melanoma in this study (NCT02272855). A total of 46 patients were enrolled in this clinical trial, and results were evaluated in 44 patients. Regarding tumor growth inhibition, the best overall response (BOR) was evaluated by Immune-Related Response Criteria at 24 weeks. BOR was 41% (irCR: 16%, irPR: 25%), clinical therapeutic efficacy was 68% (irCR + irPR + irSD), and irSD was 27%. Regarding survival rate, median progression-free survival was 19 months and median overall survival was 21.8 months. This combination showed a beneficial therapeutic effect as second-line therapy; in 20 patients, BOR was 30% (87).

## Future Directions

Over 14 years ago, HF10 was being investigated in various preclinical models, including disseminated peritoneal colon cancer, melanoma, pancreatic cancer, breast cancer, and bladder cancer. These studies have translated into successful clinical trials in different cancer types including recurrent metastatic breast cancer, recurrent HNSCC, advanced pancreatic cancer, refractory and superficial cancers, and melanoma. Although the data on HF10 in preclinical and clinical trials suggest that therapeutic applications can be developed with a high safety margin, combination therapies with either chemotherapy or immunotherapeutic agents are a promising approach in the near future. However, the ideal combination with HF10 still needs more investigation. As few OVs have shown efficacy against cancer stem cells and chemoresistant cells, more studies of HF10 against these types of cells are needed. For OVs in general, future studies must overcome physical tumor barriers that limit intravenous delivery.

## Author Contributions

IE: preparing manuscript, collecting data, designing, writing, and editing. YN: revising manuscript and providing critical considerations for manuscript design. TI: manuscript collecting data and editing. IB-V: revising manuscript and editing. MT, WZ, NMu, TM, NMi, HH, SS, BA, and YK: contributing to manuscript design and collecting data. HK: revising manuscript and providing critical considerations for manuscript design as well as final approval of the version to be published.

## Conflict of Interest Statement

Maki Tanaka is an employee of Takara Bio Inc. The remaining authors report no conflicts of interest in this work.
